# Metabolic rewiring of bacterial subpopulations governs polymyxin responses in *Acinetobacter baumannii*

**DOI:** 10.1128/msystems.00261-26

**Published:** 2026-08-07

**Authors:** Mei-Ling Han, Zhi Ying Kho, Xingjian Wang, Jinxin Zhao, Ben Finnin, Yan Zhu, Christopher K. Barlow, Darren J. Creek, Tony Velkov, Mohammad A. K. Azad, Jian Li

**Affiliations:** 1 Infection Program and Department of Microbiology, Biomedicine Discovery Institute, Monash University 214149, Clayton, Victoria, Australia; 2 Cell Therapy Pty Ltd, Melbourne, Victoria, Australia; 3 Systems Biology Centre, Tianjin Institute of Industrial Biotechnology, Chinese Academy of Sciences 165087, Tianjin, China; 4 Monash Proteomics and Metabolomics Platform, Biomedicine Discovery Institute, Monash University 161661, Clayton, Victoria, Australia; 5 Department of Biochemistry, Monash Biomedicine Discovery Institute, Monash University 161661, Clayton, Victoria, Australia; 6 Drug Delivery, Disposition and Dynamics, Monash Institute of Pharmaceutical Sciences, Monash University 588777, Parkville, Victoria, Australia; 7 Department of Pharmacology, Monash Biomedicine Discovery Institute, Monash University 534222, Clayton, Victoria, Australia; The University of Texas Southwestern Medical Center, Dallas, Texas, USA

**Keywords:** *Acinetobacter baumannii*, polymyxins, heteroresistance, cell-sorting, metabolomics, arginine metabolism, PI staining

## Abstract

Polymyxins are often last-resort antibiotics against high-priority Gram-negative pathogens, particularly *Acinetobacter baumannii*. However, most mechanistic studies to date have overlooked the heterogeneity within bacterial populations during polymyxin treatment. Using time-lapse imaging and propidium iodide (PI) staining, we observed that a subset of PI-positive (PI^+^) cells, which are traditionally considered non-viable, were capable of regrowth. This unexpected scenario promoted further investigation into the distinct metabolic responses of PI^+^ or PI-negative (PI^−^) subpopulations following polymyxin exposure. By combining a synthetic fluorescent polymyxin probe, FADDI-043, with fluorescence-activated cell sorting (FACS), we isolated PI^+^ and PI^−^ cells and profiled their metabolic responses. PI^+^ cells exhibited increased levels of phosphatidylethanolamine, likely to compensate for the severe membrane damage by polymyxins. In contrast, PI^−^ cells, where polymyxin interacted with bacterial membranes without causing extensive damage, demonstrated broader metabolic adaptations. Notably, arginine metabolism was uniquely upregulated in the PI^−^ group, and exogenous arginine supplementation conferred protection against polymyxin treatment. Collectively, this is the first study to demonstrate subpopulation-specific metabolic responses to polymyxins, highlighting dynamic and heterogeneous bacterial adaptations. Our findings underscore the importance of single-cell analysis to unravel antibiotic mechanisms and may inform novel metabolic reprogramming strategies to enhance antibiotic efficacy and minimize resistance emergence.

**IMPORTANCE:**

Multidrug-resistant *Acinetobacter baumannii* is designated a World Health Organization “critical priority” pathogen, and polymyxins remain the few effective treatment options, particularly in low- and middle-income countries. However, polymyxin heteroresistance poses a major global clinical challenge, and its mechanistic basis remains poorly defined. Most antimicrobial studies rely on population-level measurements, obscuring the biological consequences of phenotypic heterogeneity. Here, we demonstrate that a subset of polymyxin-treated, propidium iodide-positive (PI^+^) *A. baumannii* cells, typically classified as non-viable, retain the capacity to regrow. By isolating PI^+^ and PI-negative (PI^−^) subpopulations, we uncover distinct metabolic adaptations under polymyxin exposure: PI^+^ cells exhibit elevated phosphatidylethanolamine levels, whereas the PI^−^ cells activate their arginine metabolism. These findings reveal an unrecognized layer of metabolic heterogeneity underlying polymyxin exposure. Our work challenges conventional interpretations of viability assays and highlights the importance of subpopulation-resolved analyses for identifying metabolic vulnerabilities to enhance polymyxin efficacy while limiting resistance emergence.

## INTRODUCTION

The increasing prevalence of antibiotic resistance has become one of the greatest threats to modern healthcare (1). Compounding this issue, even bacteria classified as susceptible often exist as heterogeneous populations capable of surviving antibiotic treatment (2, 3). These persisting subpopulations can reduce antibiotic efficacy in clinical settings and cause treatment failure (4). Consequently, addressing phenotypic heterogeneity and understanding the distinct cellular responses of bacterial subpopulations to antibiotics are critical.

Amid these challenges, we are facing a “post-antibiotic era”. Without novel therapeutics, clinicians often use polymyxins (i.e., polymyxin B and colistin) as a last-line therapy, particularly in low- to middle-income countries. These cationic lipopeptides target lipid A of lipopolysaccharide in the outer membrane in gram-negative bacteria, disrupting membrane architecture, increasing permeability, and inducing bacterial cell death (5–7). Recent studies have shown that polymyxin treatment can induce heterogeneous bacterial subpopulations in *Acinetobacter baumannii*, a “critical priority” pathogen designated by the World Health Organization (8). Using flow cytometry and propidium iodide (PI) staining, a dye commonly employed to distinguish live and dead bacterial cells based on membrane integrity, a significant subpopulation of live cells that exhibited a non-culturable phenotype has been identified (8); however, this previous study was limited to phenotypic observations and did not investigate the physiological or metabolic cellular responses of these subpopulations under polymyxin exposure.

To address these knowledge gaps and elucidate the mechanisms of polymyxin activity and resistance, we developed a fluorescent polymyxin probe, FADDI-043 (9). Unlike the widely used dansyl-polymyxin B, which modifies the primary amines of diaminobutyric acids and damages the pharmacological activity, FADDI-043 is synthesized by selectively modifying the N-terminus of polymyxin B to preserve the antibacterial potency and nephrotoxicity of clinically used polymyxin B and colistin (9). Here, we employed FADDI-043 in combination with fluorescence-activated cell sorting (FACS) to successfully separate *A. baumannii* into two distinct subpopulations based on PI staining. Subsequent metabolomic analysis of each subpopulation revealed distinct metabolic signatures, particularly in lipid and amino acid metabolism. It is evident that *A. baumannii* survives polymyxin killing via diverse, previously unrecognized strategies that are masked in conventional bulk metabolomic analyses.

## RESULTS

### Bacterial cell sorting based on PI staining

**Fig 1:**
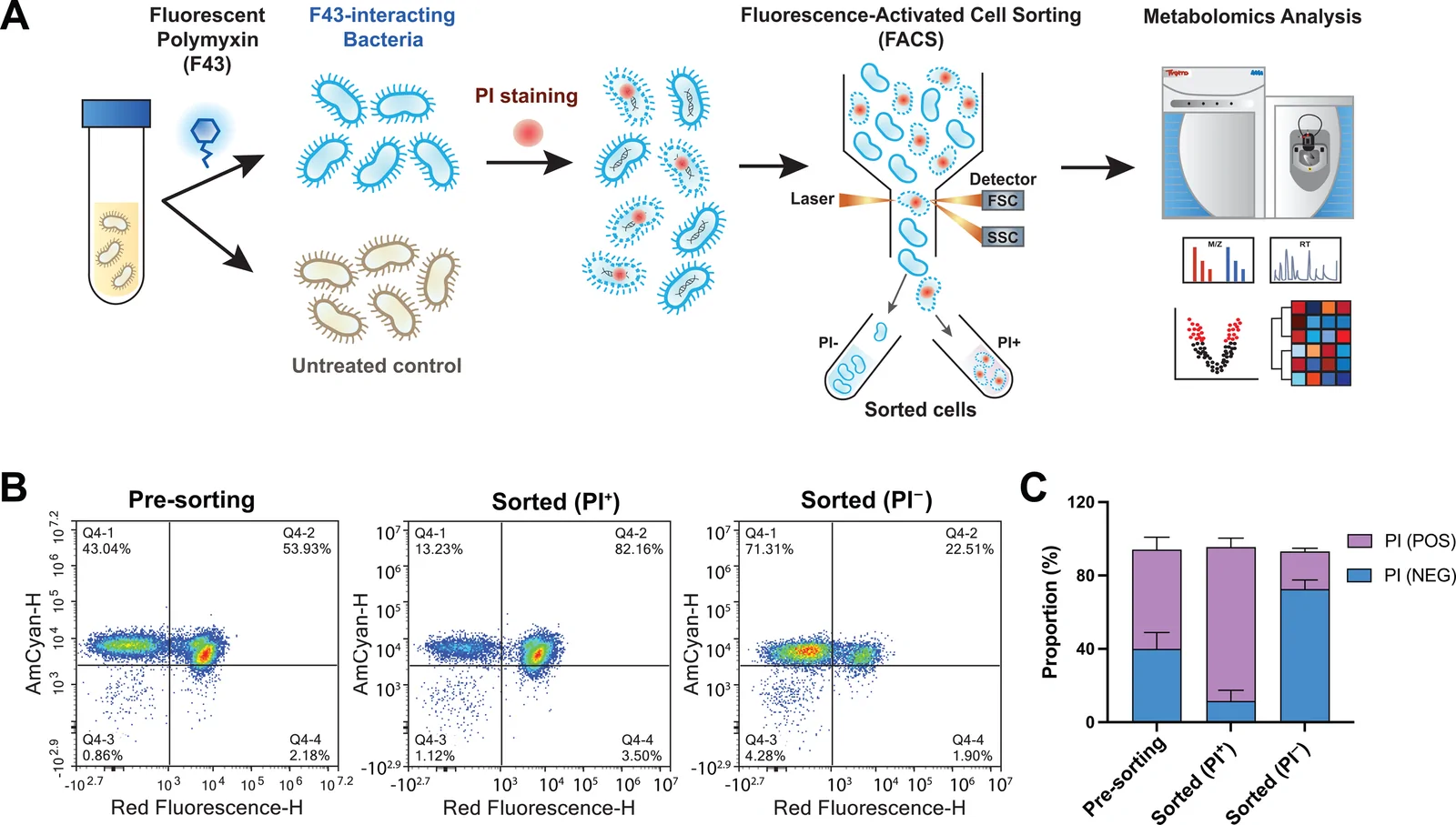
Bacterial cell sorting to separate live and dead bacterial cells for metabolomic analysis. (**A**) Workflow for bacterial cell sorting and metabolomic analysis; (**B**) FACS analysis of FADDI-043-treated bacterial cells at the stages of pre-sorting (left) and after sorting (middle and right); (**C**) proportion of PI^+^ and PI^−^ cells in each individual subpopulation (*n* = 3).

*A. baumannii* strain AB5075 was treated with polymyxin B dansyl-probe FADDI-043 at its minimum inhibitory concentration (MIC) of 8 mg/L for 1 h prior to PI staining and then subjected to flow cytometry analysis, which revealed that over 80% of the bacterial cells interacted with the probe (Fig. 1A; Fig. S1). The FADDI-043-treated bacterial cells were separated into two distinctive subpopulations, displaying PI-positive (PI^+^) (54.1% ± 6.8% of total bacterial cells) and PI-negative (PI^−^) (40.0% ± 9.0% of total bacterial cells) (Fig. 1B and C; Fig. S2). After cell sorting, we collected 7.3 log_10_ CFU/mL and 6.5 log_10_ CFU/mL of the bacterial cells in the PI^+^ and PI^−^ groups, respectively, with 83.9% ± 4.9% and 72.5% ± 5.1% of the bacterial cells in each respective group belonging to a single subpopulation (Fig. 1B and C; Fig. S2). We also validated our cell sorting efficiency using imaging microscopy to ensure the minimum contamination from another population. Clearly, both PI^+^ and PI^−^ groups exhibited a similar number of bacterial cells (blue channel), with only the PI^+^ group displaying evident PI fluorescence (red channel) (Fig. S3). In summary, our cell sorting results indicated that FADDI-043 treatment led to two distinct subpopulations in *A. baumannii* cells.

**Fig 2:**
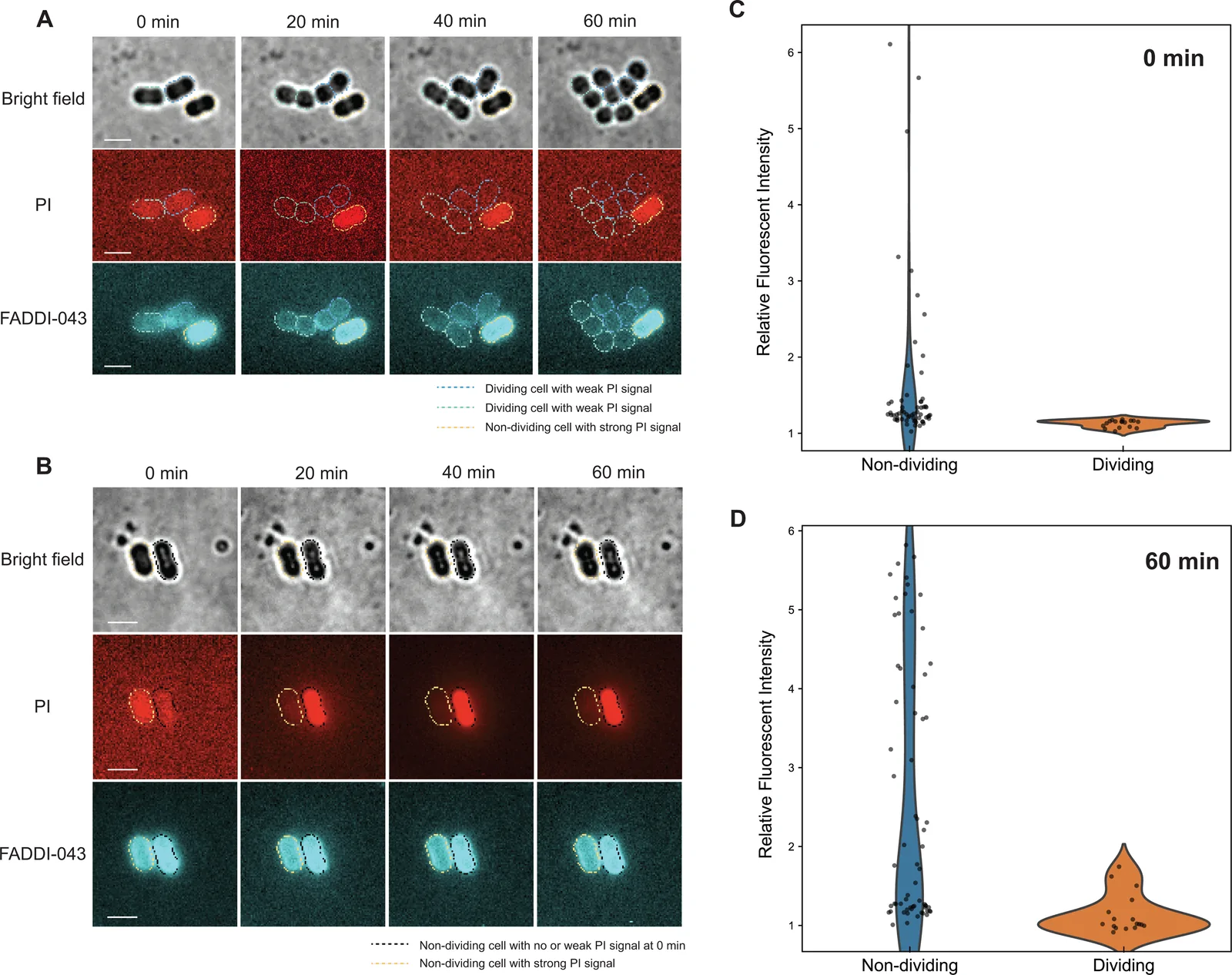
Real-time fluorescence imaging of dividing and non-dividing AB5075 cells with PI staining and 2 mg/L FADDI-043 treatment over 60 min. (**A**) PI^+^ cells displayed cell division or non-division; (**B**) PI^−^ cells displayed non-division; (**C and D**) the relative PI fluorescent intensity of non-dividing and dividing cells at (**C**) 0 min and (**D**) exposed to 2 mg/L FADDI-043 at 60 min.

Most previous studies simply classified PI^+^ bacterial cells as dead or non-dividing cells and PI^−^ cells as live or dividing cells (8, 10–12); to address this limitation, we conducted time-lapse imaging to investigate the relationship between PI signals and cell division (Fig. 2). To our surprise, in the presence of 2 mg/L FADDI-043, some AB5075 cells initially displayed weak PI signals but started dividing as early as 20 min, with their PI signals being diminished until 60 min (Fig. 2A). In contrast, several non-dividing cells displayed very weak or even no PI signals at the beginning but appeared to take up more PI at later time points (Fig. 2B). In summary, although the non-dividing cells generally showed higher PI signals compared to the dividing cells (Fig. 2C and D), there is no clear cutoff between PI fluorescence intensities and cell division, indicating the complexity of bacterial survival strategies and heterogeneous responses to polymyxin treatment.

### Metabolic variations between PI^+^ and PI^−^ bacterial cells due to polymyxin treatment

**Fig 3:**
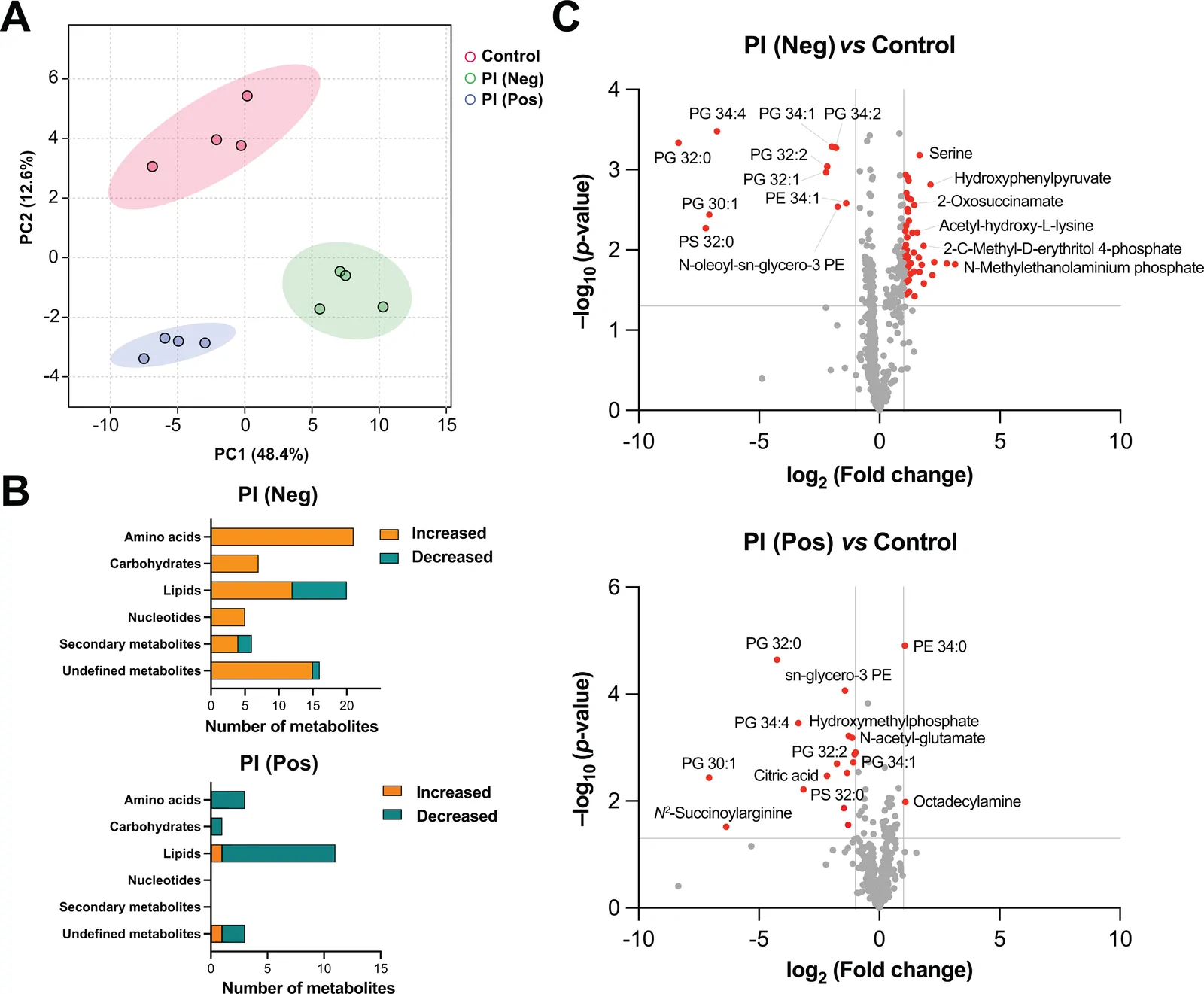
Metabolomics analysis of PI^+^ and PI^−^ bacterial cells in comparison with non-treated control. (**A**) PCA score plots of non-treated control, PI^+^ and PI^−^ bacterial groups; (**B**) number of significantly changed metabolites (log_2_ fold change > 1 or < −1 and adjusted *P*-value < 0.05) in the PI^−^ (top) and PI^+^ (bottom) groups; (**C**) volcano plots showing significantly changed metabolites in the PI^−^ (top) and PI^+^ (bottom) groups.

To investigate the heterogeneous responses of bacterial cells following exposure to polymyxins, we conducted a metabolomic study with the above sorted PI^+^ and PI^−^ bacterial cell populations and compared the metabolic changes to the respective untreated control. Metabolites were putatively identified based on accurate mass and retention time, and after filtering out unwanted metabolites (e.g., low-confidence annotations and polypeptide fragments), a total of 480 metabolites were identified primarily in the classes of amino acids, carbohydrates, lipids, and nucleotides. According to the principal component analysis (PCA), all the four replicates within each group were clustered well and were different from those of the other groups (Fig. 3A). Notably, in comparison with the untreated control samples, the PI^−^ group in which the FADDI-043 molecules did not substantially damage the membrane integrity or penetrate into the cells displayed more dramatic metabolic perturbations than in PI^+^ cells (Fig. 3B and C; Fig. S4). In detail, a total of 71 (60/11, increased/decreased) metabolites were significantly changed (log_2_ fold change [FC] > 1 or < −1; adjusted *P*-value < 0.05) in the PI^−^ group, while only 17 (2/15, increased/decreased) were differentially perturbed in the PI^+^ group (Fig. 3B). Particularly, 21 amino acid, 5 carbohydrate, 12 lipid, and 5 nucleotide metabolites were significantly increased, and 8 lipids were decreased in the PI^−^ group (Fig. 3B, upper). On the other hand, the PI^+^ group mainly displayed decreased abundance of 10 lipid metabolites (Fig. 3B, lower). It is notable that most of the decreased metabolites in the PI^+^ group were phosphatidylglycerol (PG), a major membrane lipid component which contains negative charge, with all detected PG (PG 30:1, 32:0, 32:2, 34:1, 34:2, and 34:4) showing decreased abundance in both the PI^+^ and PI^−^ groups (log_2_FC > 1 or < −1; *P*-value < 0.05; Fig. 3C).

### Uniquely affected metabolic changes in the PI^−^ group

A number of amino acid metabolites were uniquely increased in the PI^−^ group, including several key metabolites of arginine biosynthesis and degradation (i.e., L-arginine, *N*^2^-succinyl-L-arginine, *N*^2^-succinyl-L-ornithine, *N*-acetyl-L-glutamate 5-semialdehyde, and *N*-acetyl-L-citrulline). In addition, several other amino acid metabolites, including phenylalanine (log_2_FC = 1.10), tyrosine (log_2_FC = 1.03), and phenylpyruvate (log_2_FC = 1.42) in phenylalanine biosynthesis and methionine (log_2_FC = 1.07), tryptophan (log_2_FC = 1.18), asparagine (log_2_FC = 1.01), histidine (log_2_FC = 1.36), leucine (log_2_FC = 1.02), and serine (log_2_FC = 1.66), were significantly increased in the PI^−^ group, while remaining unperturbed in the PI^+^ group; a similar pattern was also observed with several metabolites in nucleotide metabolism, such as cytidine (log_2_FC = 1.06), hypoxanthine (log_2_FC = 1.11), and deoxyadenosine (log_2_FC = 1.07) (Table S1). Given that most of these metabolites were accumulated to a similar extent (i.e., log_2_FC ≈ 1), it is likely that the FADDI-043-treated bacterial cells paused their protein and RNA synthesis, thus accumulating amino acid and nucleotide bases.

Apart from the significantly affected amino acid metabolism in the PI^−^ group, two metabolites, namely, *N*-acetylneuraminate (also called sialic acid) (log_2_FC = 1.00) and CMP-3-deoxy-D-manno-octulosonate (CMP-KDO; log_2_FC = 1.18), were also only increased in the PI^−^ group (Table S1). Given that sialic acid is found in the lipopolysaccharide (LPS) O-antigen of diverse Gram-negative species and CMP-KDO serves as a key intermediate to form rough-type LPS (13), their increase suggests that the biosynthesis of LPS was affected due to FADDI-043 treatment.

**Fig 4:**
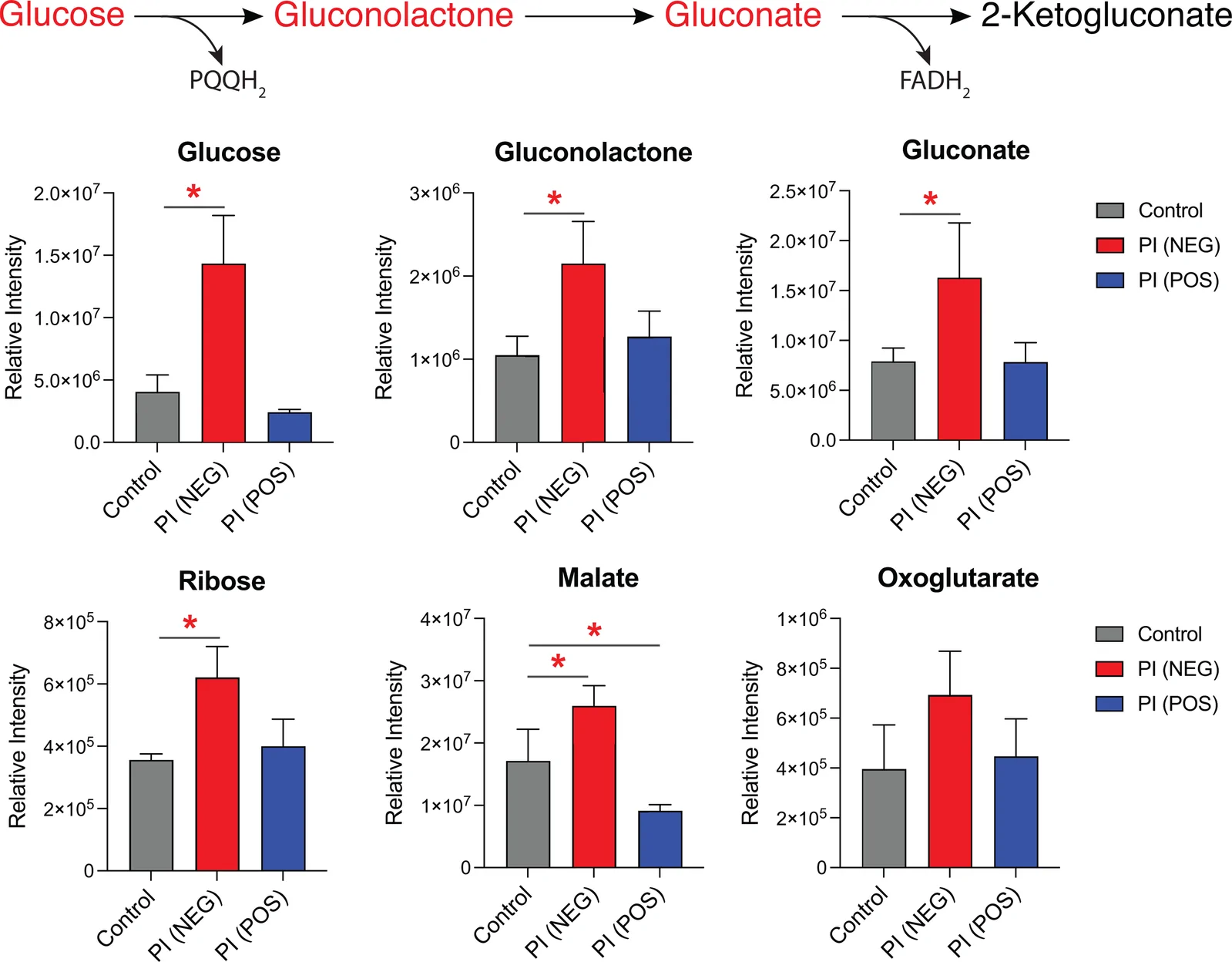
Metabolic changes in glucose oxidation. Metabolite name in red: significantly increased in the PI^−^ group. Relative intensity refers to peak height measured by LC-MS. Error bar stands for standard deviation. *P < 0.05 (*n* = 4). Abbreviations: PQQH_2_, reduced pyrroloquinoline quinone; FADH_2_, reduced flavin adenine dinucleotide.

Several carbohydrate metabolites were also significantly altered. Using authentic standards, we confirmed that glucose (log_2_FC = 1.82), gluconolactone (log_2_FC = 1.04), and gluconate (log_2_FC = 1.05) were significantly increased in the PI^−^ group, but no significant change was observed in the PI^+^ group (Fig. 4). In addition, several other carbohydrate-related metabolites showed modest increases in the PI^−^ group, including ribose (log_2_FC = 0.80) from the pentose phosphate pathway and oxoglutarate (log_2_FC = 0.81) and malate (log_2_FC = 0.60) from the citric acid cycle. Given that both gluconolactone and gluconate are intermediates of glucose oxidation and that gluconate degradation links directly to the pentose phosphate and citric acid cycle pathways (14), these increases collectively indicate that FADDI-043 treatment enhanced glucose degradation.

### Uniquely perturbed arginine metabolism in the PI^−^ group

**Fig 5:**
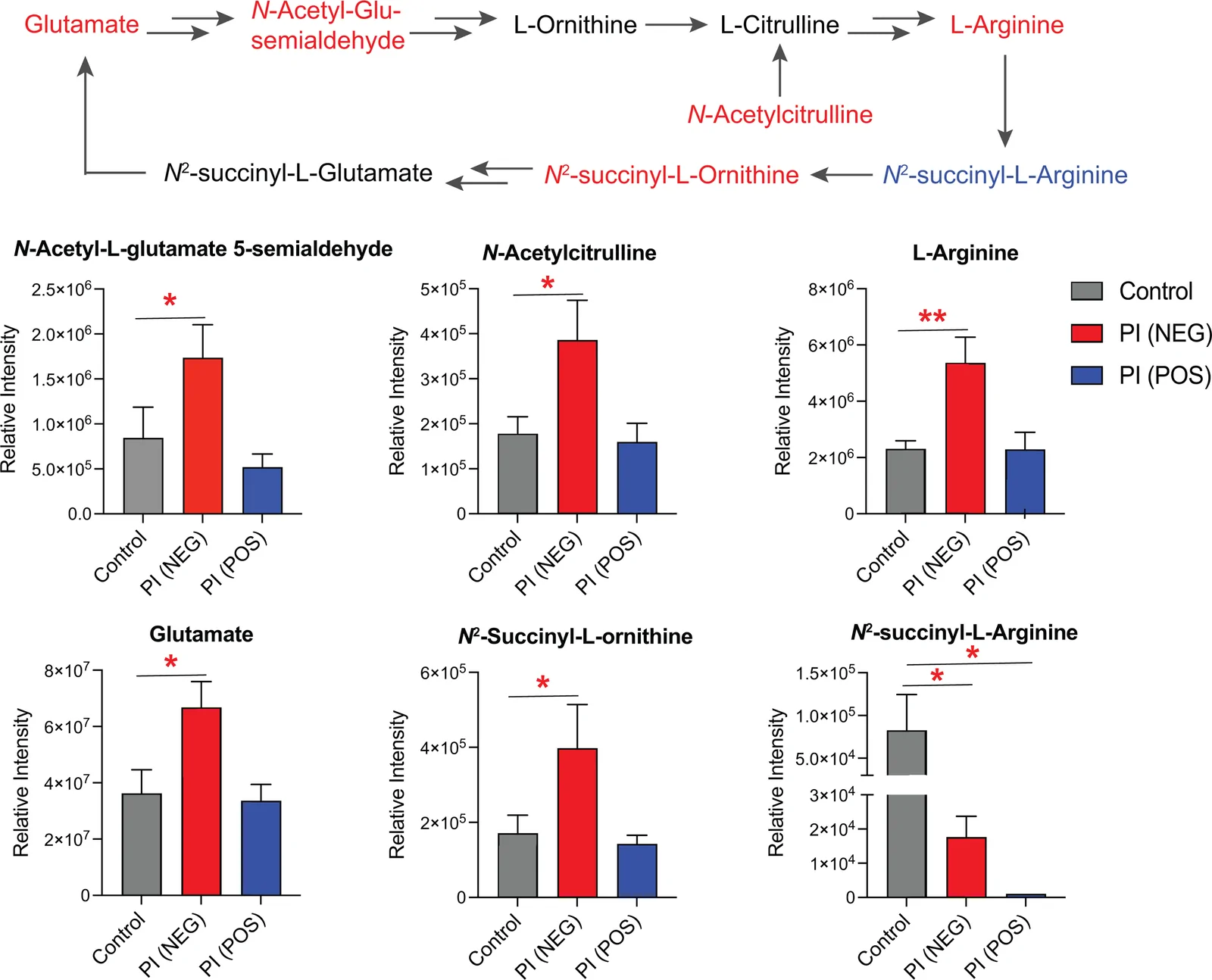
Metabolic changes associated with arginine synthesis and degradation. Metabolite name in red: significantly increased in the PI^−^ group; blue: significantly decreased in both PI^−^ and PI^+^ groups. Relative intensity refers to peak height measured by LC-MS. Error bar stands for standard deviation. *P < 0.05, **P < 0.01 (*n* = 4).

Among the uniquely perturbed metabolism in the PI^−^ group, the most significant change was observed in the arginine biosynthesis and degradation pathways (Fig. 5). Specifically, compared to the untreated control, arginine (log_2_FC = 1.22), glutamate (log_2_FC = 0.88), *N*-acetyl-L-glutamate 5-semialdehyde (log_2_FC = 1.04), *N*-acetyl-citrulline (log_2_FC = 1.12), and *N*^2^-succinyl-L-ornithine (log_2_FC = 1.22) were all significantly increased in the PI^−^ group, while without significant change in the PI^+^ group (Fig. 5). Consistently, a few other metabolites involved in arginine metabolism, namely, *N*-acetyl-L-glutamate 5-semialdehyde (log_2_FC = 1.04 and −0.70 in the PI^−^ and PI^+^ groups, respectively), hydroxy-acetyl-L-arginine (log_2_FC = 1.29 and −0.72), and beta-alanyl-L-arginine (log_2_FC = 1.09 and −0.62) (Fig. 4), were significantly increased in the PI^−^ group but slightly decreased in the PI^+^ group. However, a metabolite which was putatively annotated as *N*^2^-succinyl-L-arginine, an important intermediate in the arginine degradation pathway, was significantly depleted in both PI^+^ (log_2_FC = −6.37) and PI^−^ (log_2_FC = −2.23) groups (Fig. 5). These results indicated that the PI^−^ cells are likely to activate their arginine metabolism to compensate for membrane damage.

**Fig 6:**
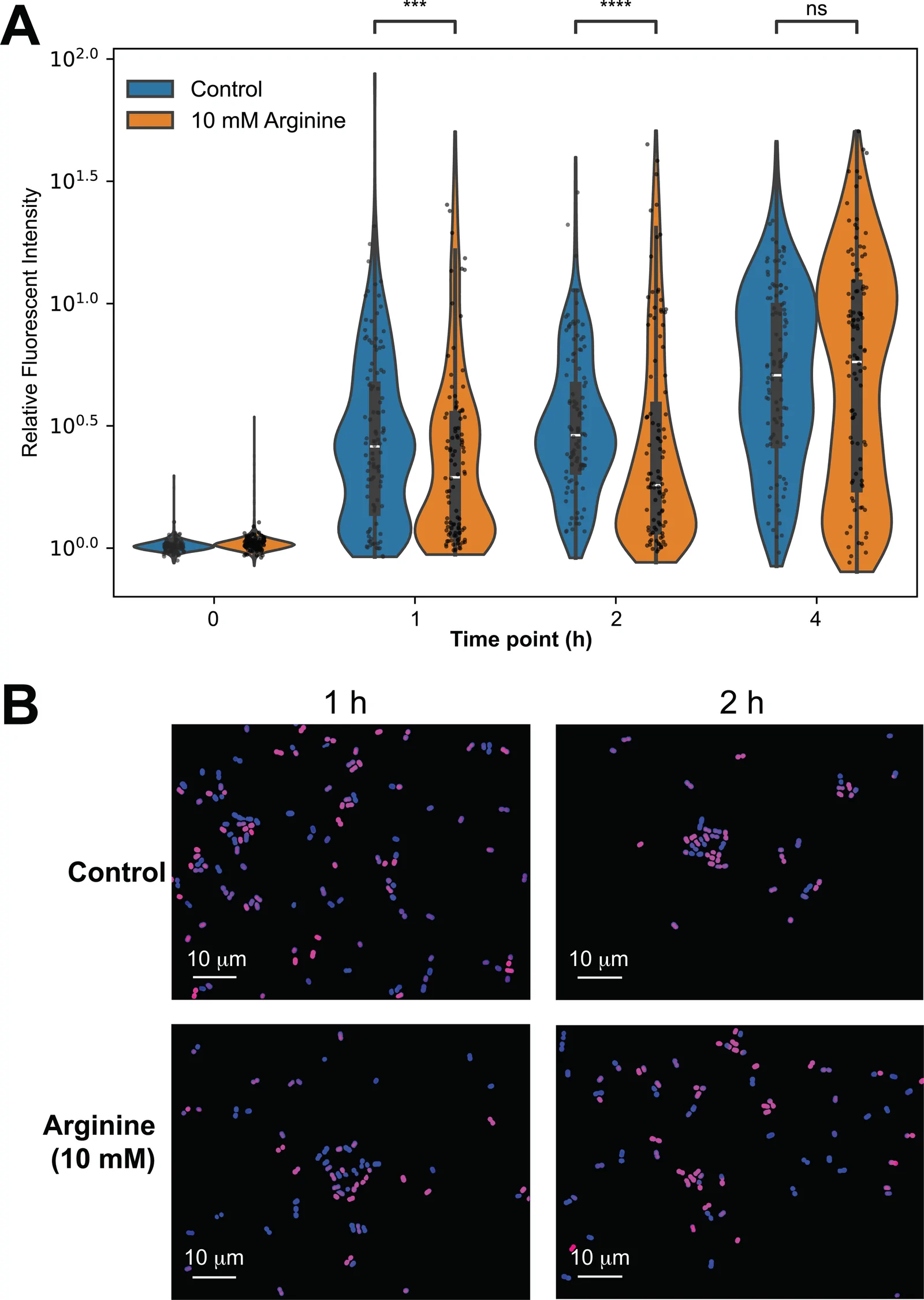
The presence of 10 mM arginine decreased PI uptake in 8 mg/L FADDI-043-treated AB5075. (**A**) The relative fluorescent intensity of PI staining in the FADDI-043-treated AB5075 cells with or without 10 mM arginine for 1, 2, and 4 h; (**B**) overlaid FADDI-043 (blue) and PI (pink) fluorescence imaging in AB5075 at 1 and 2 h. ***P < 0.001, ****P < 0.00001, ns: not significant.

To test this hypothesis, we performed a further fluorescence imaging study with the supplementation of 10 mM arginine. The results showed that exogenous arginine significantly reduced the overall relative intensity of PI staining by 40.6% (*P* < 0.001) and 57.1% (*P* < 0.0001) at 1 and 2 h, respectively (Fig. 6). Notably, at 2 h, arginine appeared to minimize the differentiation of subpopulations induced by FADDI-043 treatment at 8 mg/L. These findings provide clear evidence that arginine metabolism plays a critical role in protecting bacterial cells against polymyxin-mediated membrane damage.

### Phosphatidylethanolamine was uniquely increased in the PI^+^ group

**Fig 7:**
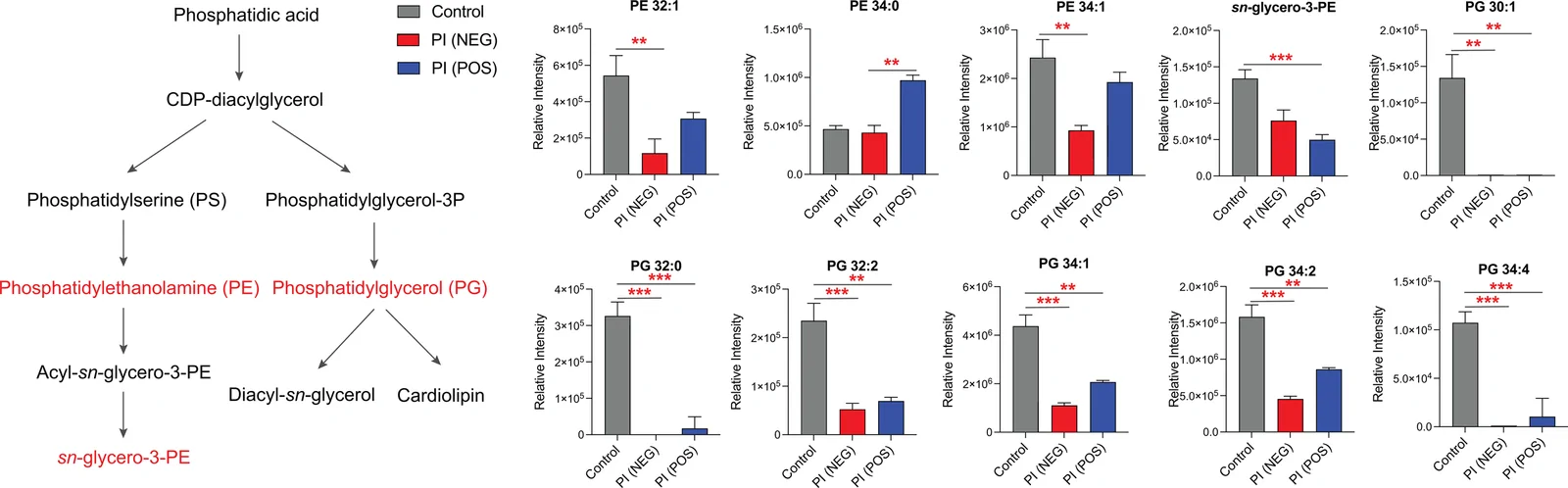
Metabolic changes in glycerophospholipid metabolism. Significantly changed lipid classes are shown in red. *P < 0.05, **P < 0.01, ***P < 0.001 (*n* = 4).

Pathway enrichment analysis with the above differentially changed metabolites highlighted that essential metabolism, including glycerophospholipid metabolism, arginine synthesis, arginine and proline metabolism, and D-amino acid metabolism, were significantly affected in response to FADDI-043 treatment (Fig. S5). Notably, bacterial glycerophospholipid metabolism was affected in both the PI^+^ (FADDI-043 damaged bacterial membranes) and PI^−^ (without significantly changing membrane integrity) groups, which was consistent with the well-established membrane-targeting mechanisms of polymyxins (Fig. 7). Specifically, a major membrane phospholipid, phosphatidylglycerol (PG), including PG 30:1 (only detected in control but absent in both PI^+^ and PI^−^ groups), PG 32:0 (log_2_FC = −4.25/−8.35; PI^+^/PI^−^), PG 34:1 (log_2_FC = −1.08/−1.98), PG 34:2 (log_2_FC = −0.88/−1.80), PG 32:2 (log_2_FC = −1.76/−2.17), and PG 34:4 (log_2_FC = −3.36/−6.75), was significantly decreased in both PI^+^ and PI^−^ groups compared to their respective untreated controls. In contrast, another major membrane lipid component, phosphatidylethanolamine (PE), including PE 34:1 (log_2_FC = −1.39) and PE 32:1 (log_2_FC = −2.22), was mainly decreased in the PI^−^ group with only minor change (log_2_FC > −1) in the PI^+^ group. Notably, PE 34:0 (log_2_FC = 1.06) was uniquely increased in the PI^+^ group but not changed in the PI^−^ group (Fig. 7). Consistently, an intermediate *sn*-glycero-3-phosphoethanolamine involved in PE degradation decreased (log_2_FC = −1.43) in the PI^+^ group. The decreased PE degradation may link to PE accumulation, consistent with increased level of PE 34:0 in the PI^+^ group, indicating that the bacterial cells synthesize more PE to compensate for the damaged membrane due to polymyxins.

## DISCUSSION

To the best of our knowledge, this study is the first to integrate cell sorting with metabolomic analysis to investigate heterogeneous bacterial responses to antibiotic treatment. By combining a fluorescent polymyxin probe, FACS, time-lapse imaging, and metabolomics, we dissect heterogeneous bacterial responses to polymyxin exposure at the level of distinct bacterial subpopulations. Our results reveal that *A. baumannii* survives polymyxin stress through multiple, previously unappreciated strategies that are masked in conventional bulk metabolomic analyses. Together, this study establishes an experimental framework for advancing antimicrobial systems pharmacology through the analysis of individual bacterial cells.

First, our data demonstrate that PI staining does not strictly define bacterial viability under polymyxin stress. In contrast to the prevailing literature that classifies PI^+^ bacterial cells as non-dividing or dead (8, 10–12), our time-lapse imaging results clearly demonstrated that PI positivity is not synonymous with loss of viability. A subset of PI^+^ cells retains the capacity to divide, while some PI^−^ cells later accumulated PI without undergoing division. Importantly, there was no clear fluorescence threshold separating dividing from non-dividing cells. These findings fundamentally challenge the conventional interpretation of PI staining in bacterial cells and highlight the dynamic and reversible nature of polymyxin-induced membrane perturbation, bacterial survival strategies, and post-treatment metabolic adaptation. Rather than representing terminal damage, PI uptake may reflect transient or localized membrane defects that can be repaired, at least in a subset of cells. This heterogeneity provides a plausible explanation for bacterial survival and regrowth following polymyxin treatment despite extensive membrane targeting.

Second, our metabolomic analysis further revealed that the PI^+^ and PI^−^ subpopulations exhibited distinct metabolic responses, particularly in lipid and amino acid metabolism, indicating that bacterial adaptation varies with the extent of membrane damage. Importantly, the cell-sorting approach also addresses a key limitation of bulk metabolomics, which did not differentiate between live, dead, or membrane-damaged bacterial cells, and underestimates adaptive responses to antibiotics. In bulk metabolomics studies, the metabolic profiles are often confounded by the mixture of multiple subpopulations, obscuring strikingly divergent metabolomic responses (15). By separating bacterial subpopulations based on membrane integrity, we were able to capture more accurate intracellular metabolic signatures from viable cells. Notably, the PI^−^ subpopulation, despite showing no detectable membrane permeabilization, exhibited far more extensive metabolic remodeling than the PI^+^ group. This observation suggests that the absence of PI uptake does not equate to minimal polymyxin stress; rather, these cells mounted an active and metabolically intensive adaptive response.

Among metabolomic studies conducted on the entire bacterial populations, polymyxins have been shown to significantly disrupt phospholipid and fatty acid metabolism, as well as peptidoglycan and LPS biogenesis pathways, reflecting their membrane-targeting mechanisms (15–17). This membrane-disrupting activity is particularly important for the synergistic interaction between polymyxins and carbapenems in the treatment of multidrug-resistant *A. baumannii* infections (18). Consistent with this mechanism, previous metabolomics studies have demonstrated time-dependent metabolic responses to polymyxin-carbapenem combination therapy, characterized by early perturbations in outer membrane and cell wall biogenesis, followed by substantial disruptions in central carbon, amino acid, and nucleotide metabolism during prolonged exposure (16, 19). In the present study, we observed similar reductions in membrane phospholipids, especially phosphatidylglycerol, in both PI^+^ and PI^−^ groups, further confirming the direct impact of polymyxin on the integrity of gram-negative bacterial membrane. Moreover, unlike consistent PG loss in both PI^+^ and PI^−^ cells, PE remodeling was subpopulation-specific. The PI^+^ group uniquely accumulated PE 34:0, accompanied by reduced PE degradation intermediates, suggesting active membrane repair through PE synthesis. As a zwitterionic lipid, PE plays a role in modulating membrane curvature (20), acting as a lipochaperone (21), and supporting membrane protein topogenesis (22). Its upregulation may reinforce membrane stability, enhance defense against polymyxins, and facilitate bacterial regrowth. The PI^−^ group, however, showed a net decrease in several PE species, indicating that these cells rely less on structural lipid replacement and more on metabolic and biochemical adaptation to maintain membrane integrity. This dichotomy highlights two fundamentally different survival strategies: structural membrane repair in PI^+^ cells versus metabolic reprogramming in PI^−^ cells. This inference is also supported by much broader metabolic changes in the PI^−^ subpopulation, particularly in amino acid and carbohydrate metabolism. The accumulation of amino acids and nucleotide bases in the PI^−^ group, with relatively uniform log_2_ fold changes around +1, likely reflects a global slowdown or arrest of macromolecular synthesis. Such metabolic pausing may represent a protective strategy, allowing cells to conserve resources while redirecting energy toward stress adaptation and membrane repair (23). In contrast, the PI^+^ group showed comparatively limited metabolic flexibility, dominated by lipid depletion, consistent with direct membrane damage overwhelming adaptive capacity.

Thirdly, activation of arginine metabolism protects against polymyxin-mediated membrane damage. Arginine metabolism was significantly upregulated only in the PI^−^ group, indicating that intact cells may activate arginine catabolism as a stress response to mitigate early membrane damage (24). The coordinated increase in arginine, glutamate-derived intermediates, and succinylated arginine pathway metabolites strongly indicates an active upregulation of arginine biosynthesis and turnover (25). This aligns with our previous findings that arginine catabolism supports membrane phospholipid remodeling and promotes survival in polymyxin-dependent resistant bacterial isolates (26). Arginine metabolism has multiple functions relevant to polymyxin stress. Arginine catabolism can generate ATP, provide precursors for polyamine synthesis, and modulate intracellular pH, all of which may enhance membrane stability and stress tolerance (27). Importantly, exogenous arginine supplementation significantly reduced PI staining and diminished subpopulation heterogeneity, providing functional validation that arginine metabolism directly contributes to protection against polymyxin-induced membrane disruption. The depletion of *N*^2^-succinyl-L-arginine in both PI^−^ and PI^+^ cells, despite upstream accumulation of arginine intermediates, suggests enhanced flux through downstream degradation pathways, potentially to fuel rapid energy production or stress-responsive biosynthesis. Collectively, these findings position arginine metabolism as a critical determinant of polymyxin tolerance rather than merely a bystander metabolic response.

Fourthly, we discovered differential remodeling of LPS and central carbon metabolism in PI^−^ cells. The selective increase in CMP-KDO and *N*-acetylneuraminate in the PI^−^ group indicates perturbation of LPS biosynthesis (28). Given that polymyxin targets lipid A via electrostatic interactions (6), enhanced LPS turnover or remodeling may mitigate its disruptive effects. This aligns with previous observations that alterations in LPS composition contribute to polymyxin resistance and tolerance (13). In parallel, the accumulation of glucose, gluconate, and gluconolactone, together with modest increases in pentose phosphate pathway and TCA cycle intermediates, indicates enhanced glucose oxidation in PI^−^ cells (29, 30). The pentose phosphate pathway provides NADPH, which is essential for reductive biosynthesis and oxidative stress defense, further supporting a coordinated metabolic strategy in PI^−^ cells aimed at survival rather than growth (31).

Together, our findings demonstrate that bacterial survival under polymyxin stress is not binary but involves a spectrum of adaptive states. PI^−^ cells engage extensive metabolic reprogramming to pre-empt membrane damage, whereas PI^+^ cells attempt post-damage membrane repair (32). These heterogeneous strategies likely coexist in clinical infections and contribute to treatment failure and bacterial persistence. Importantly, our study underscores the limitations of bulk metabolomics and conventional viability assays in antimicrobial research. Single-cell resolved approaches are essential to capture the complexity of bacterial stress responses and may uncover previously unrecognized metabolic vulnerabilities. As single-cell metabolomics continues to advance, future studies will be better positioned to address the key research questions arising from this work, particularly whether the observed metabolic changes, such as altered arginine metabolism, are induced by antibiotic exposure or instead reflect pre-existing metabolic heterogeneity that influences individual bacterial cell fate under antimicrobial stress.

Beyond polymyxins, extending this single-cell approach to emerging classes of antibiotics that target bacterial membrane biogenesis may provide further insights into phenotypic heterogeneity and mechanisms underlying treatment failure. In addition, applying this framework across multiple members of the *A. baumannii* complex would help determine whether the heterogeneous metabolic adaptations discovered in this study are conserved across species or instead represent species-specific responses. For instance, zosurabalpin, a novel macrocyclic peptide that disrupts LPS transport in *Acinetobacter* spp. (33), represents a promising candidate for investigating how membrane-targeting antibiotics shape bacterial phenotypic and metabolic heterogeneity at the single-cell level.

In conclusion, this study demonstrates, for the first time, that bacterial heterogeneity is a critical determinant of cellular responses to polymyxin treatment. We address a major knowledge gap by revealing that key metabolic changes were obscured in conventional entire-population analysis. By separating subpopulations using cell sorting, we provide a more accurate and detailed view of bacterial adaptation to antibiotic pressure at the subpopulation level. These findings reveal novel mechanistic insights into polymyxin-induced metabolic reprogramming and highlight the importance of cellular heterogeneity in antimicrobial research. Furthermore, our approach identifies potential metabolic vulnerabilities that may be exploited to enhance polymyxin efficacy and combat antibiotic resistance.

## MATERIALS AND METHODS

### Chemical and reagents

The fluorescent polymyxin probe FADDI-043 was synthesized in our laboratory as previously mentioned (9). Its stock solution was prepared using Milli-Q water (Millipore Australia, North Ryde, New South Wales, Australia) and filtered through 0.22-μm syringe filters (Sartorius, Melbourne, VIC, Australia). All solvents and reagents used in the liquid chromatography-mass spectrometry (LC-MS) analysis were purchased from Thermo Fisher (USA) at LC or MS grade.

### Bacterial cell sorting

Prior to experiments, the model strain *A. baumannii* AB5075 was sub-cultured onto a Mueller-Hinton agar plate at 37°C for 16 to 18 h. A single colony was then inoculated into 10 mL of cation-adjusted Mueller-Hinton broth (CAMHB; Oxoid) and incubated at 37°C overnight with shaking at 150 rpm. The overnight culture was diluted 1:100 in fresh CAMHB media and then grown to mid-logarithmic phase at an optical density at 600 nm (OD600) of 0.50 (~10^8^ CFU/mL). The bacterial culture was then treated with FADDI-043 at 8 mg/L (1× MIC) for 1 h, with untreated bacterial culture serving as a control sample. Each group consisted of four biological replicates derived from four individual bacterial colonies. Following treatment, the polymyxin-treated bacterial culture was subjected to FACS using a MACSQuant Tyto Cell Sorter to exclude non-interacting cells (34). Propidium iodide (PI) was used to assess membrane integrity, allowing differentiation between PI^+^ and PI^−^ bacterial populations. These subpopulations were separately collected into sterile, closed MACSQuant Tyto Cartridges. Untreated control samples were also stained with PI and sorted to account for potential effects associated with PI staining or the cell sorting process. Subsequently, fluorescence imaging and FACS analyses were performed to confirm the proportion of PI^+^ or PI^−^ bacterial cells within each group. The experimental workflow is shown in Fig. 1A.

### Time-lapse imaging

Time-lapse imaging was performed on bacterial cells loaded onto a CAMHB agarose gel pad containing 2.5 mg/L PI, prepared according to a modified protocol from the literature (35). The gel pad was equilibrated in a 37°C warm room prior to use. Mid-log phase bacterial cultures were exposed to 2 mg/L FADDI-043 (0.25×  MIC) for 5 min before being transferred onto the agarose gel surface. The gel pad was then sealed within an Ibidi Glass Bottom μ-Dish and incubated at 37°C using an Ibidi Heating System. Imaging was performed using a Leica DMi8 wide-field microscope equipped with a 100 × oil immersion objective (numerical aperture  =  1.32). Three channels were acquired: transmission for morphology, DAPI (excitation: 325–375 nm, emission: 435–485 nm) for FADDI-043 fluorescence, and RHOD (excitation: 541–551 nm, emission: 565–605 nm) for PI fluorescence. Images were acquired at 10-min intervals for a total duration of 1 h. Time-lapse data sets were processed using FIJI with the TrackMate 7 plugin (36), which incorporates Cellpose for single-cell segmentation (37), followed by tracking. Further image processing and downstream analysis were performed using custom Python scripts.

To validate the effect of arginine on PI uptake, mid-log phase bacterial cultures were pre-treated with 10 mM arginine for 10 min, with dH_2_O serving as the control. The culture was then exposed to 8 mg/L FADDI-043 (1×  MIC) for 1, 2, and 4 h, followed by PI staining at room temperature for 10 min. The stained samples were centrifuged at 5,000 ×  *g* for 5 min and resuspended in saline. A 0.3-μL aliquot was transferred onto an agarose gel pad and subjected to fluorescence imaging using the same Leica DMi8 microscope. Instrumental settings and image processing parameters were consistent with those described above.

### Sample preparation for metabolomic study

Cellular metabolites were extracted from the above sorted PI^+^ and PI^−^ groups together with the non-treated control using a previously optimized method with slight modifications (38). Briefly, the normalized bacterial cells at a total count of 10^6.5–7.2^ were rapidly quenched in a dry ice/ethanol bath for ~30 s to stop the metabolic processes. After washing twice with 2 mL ice-cold 0.9% NaCl, cell pellets were transferred to 1.5-mL Eppendorf tubes and resuspended in 200 μL chloroform/methanol/water (CMW, 1:3:0.9, vol/vol) containing 1 μM generic internal standards (CHAPS, CAPS, PIPES, and TRIS). This was followed by dipping the sample tubes in liquid nitrogen and thawing on ice to ensure cell lysis and metabolite release. The extracted samples were vortexed thoroughly and placed on ice for 15 min before being centrifuged at 14,000 × *g* for 10 min. The particle-free supernatant was collected into LC-MS vials for metabolomics analysis.

### Metabolomics analysis using LC-MS

The metabolomic study was conducted at the Monash Proteomics and Metabolomics Platform (Monash University, Australia). A hydrophilic interaction liquid chromatography (HILIC) coupled to high-resolution mass spectrometry was employed to detect intracellular metabolites. Samples were analyzed on a Dionex U3000 high-performance liquid chromatography system in tandem with a Q-Exactive Orbitrap Mass Spectrometer (Thermo Fisher) in both positive and negative modes with a resolution at 35,000. As previously described (38), samples were maintained at 4°C and eluted through a ZIC-pHILIC column (5 µm, polymeric, 150 × 4.6 mm; SeQuant, Merck) using aqueous phase (A) containing 20 mM ammonium carbonate and organic phase (B) containing acetonitrile. Following a linear gradient and a flow rate of 0.3 mL/min, mobile phase B was decreased from 80% to 50% over 15 min and then to 5% over the next 3 min. Mobile phase B was maintained at 5% for 3 min and then increased back to 80% during the next 3 min, and the LC system was re-equilibrated at 80% B for 8 min prior to the next run. All samples were analyzed within a single LC-MS batch to avoid variations. Pooled quality control samples were included to evaluate analyte stability, and a metabolite standard library consisting of approximately 400 authentic metabolite standards was analyzed within the same batch to aid in metabolite identification.

### Data processing and bioinformatic analysis

The metabolomics data analysis was conducted within the IDEOM workflow as previously reported (39). Briefly, raw data were first converted to mzXML and then peakML files which were subjected to MZmatch for data combination and peak annotation (40). The MZmatch data were then imported into IDEOM for metabolite identification, which was based on both mass values (<3 ppm) and retention times (<5% of variation for the metabolites with authentic standards). The standard library was employed to predict the retention time of all metabolites in the database based on a retention time calibration curve, thus enhancing the identification confidence (within 50% window for the predicted retention time). Statistical analyses were conducted in R package and MetaboAnalyst 5.0 (41) with peak intensities being normalized by the median, log transformed, and auto-scaled. Multivariate statistical analysis such as principal component analysis (PCA) was applied to assess the global metabolic profiles, and univariate statistics such as Student's *t*-test were used to identify significantly changed metabolites in both PI^+^ and PI^−^ groups in response to FADDI-043, compared to the non-treated control. Metabolites displaying a fold change of ≥2 (adjusted *P*-value < 0.05) were further analyzed and subjected to pathway analysis based on Kyoto Encyclopedia of Genes and Genomes (42) pathway and BioCyc (43).

## Data Availability

The imaging data are freely available upon request, and the metabolomics data have been deposited in the public repository MetaboLights (https://www.ebi.ac.uk/metabolights/) (European Bioinformatics Institutes) with accession number MTBLS14745.

## SUPPLEMENTAL MATERIAL

Supplemental material msystems.00261-26-s0001.docx Fig. S1–S5; Table S1.

ASM does not own the copyrights to Supplemental Material that may be linked to, or accessed through, an article. The authors have granted ASM a non-exclusive, world-wide license to publish the Supplemental Material files. Please contact the corresponding author directly for reuse.
