# Supplementary material for: Metabolic rewiring of bacterial subpopulations governs polymyxin responses in Acinetobacter baumannii
Source: mSystems. 2026 Aug 7;11(9):e00261-26. doi: 10.1128/msystems.00261-26 (PMC13595966; doi:10.1128/msystems.00261-26)
Supplement: Supplementary file 1 [file msystems.00261-26-s0001.docx]

**Supplementary Materials**

**Metabolic Rewiring of Bacterial Subpopulations Governs Polymyxin Responses in *Acinetobacter baumannii***

**Authors**

Mei-Ling Han^1^, Zhi Ying Kho^1^, Xingjian Wang^1^, Jinxin Zhao^1^, Ben Finnin^2^, Yan Zhu^3^, Christopher K. Barlow^4,5^, Darren J. Creek^4,6^, Tony Velkov^7^, Mohammad A. K. Azad^1^†, Jian Li^1*^

**Affiliations**

^1^Infection Program and Department of Microbiology, Biomedicine Discovery Institute, Monash University, Clayton, Victoria 3800, Australia.

^2^Cell Therapy Pty Ltd, Melbourne Victoria 3000, Australia.

^3^Systems Biology Centre, Tianjin Institute of Industrial Biotechnology, Chinese Academy of Sciences, Tianjin 300308, China.

^4^Monash Proteomics and Metabolomics Platform, Biomedicine Discovery Institute, Monash University, Clayton, Victoria 3800, Australia.

^5^Department of Biochemistry, Monash Biomedicine Discovery Institute, Monash University, Clayton, Victoria, Australia.

^6^Drug Delivery, Disposition and Dynamics, Monash Institute of Pharmaceutical Sciences, Monash University, 381 Royal Parade, Parkville, Victoria 3052, Australia.

^7^Department of Pharmacology, Monash Biomedicine Discovery Institute, Monash University, Clayton, Victoria, Australia.

^†^Present address: 5/5 Nuyts Street, Red Hill, ACT 2603, Australia.

*Address correspondence to: Jian Li; [jian.li@monash.edu](mailto:jian.li@monash.edu) (J.L.)

**Fig S1.** FACS analysis of AB5075 bacterial cells before treatment (left), after 8 mg/L FADDI-043 (F43) treatment (middle), and after both FADDI-043 treatment and PI staining (right). F43: FADDI-043.

**Fig S2.** FACS analysis of AB5075 bacterial cells under 8 mg/L FADDI-043 treatment at the stages of pre-sorting (left) and after sorting (middle and right) in the (**A**) second and (**B**) third replicates. This figure is associated with main **Fig. 1**, in which the first replicate was displayed.

**Fig S3.** Fluorescence imaging of pre-sorting (top) and sorted bacterial cells displaying PI^+^ (center) and PI^–^ (bottom). Scale bar = 10 μm.­­

**Fig S4.** Heatmap showing metabolic changes in PI^+^ and PI^–^ groups compared to the nontreated control (n = 4).

**Fig S5.** Pathway enrichment analysis of significantly changed metabolites in the PI^–^ group compared to nontreated control.

**Table S1.** Significantly perturbed metabolites in both PI^+^ and PI^–^ groups or each individual group.

| **Map** | **Putative metabolite** | **Mass** | **RT** | **FORMULA** | **log_2_FC**  **(PI^–^ *vs* control)** | **log_2_FC (PI^+^ *vs* control)** | | **Adjusted *p*-value** |
| --- | --- | --- | --- | --- | --- | --- | --- | --- |
| **Commonly Changed Metabolites in both PI^+^ and PI^–^ groups** | | | | | | | | |
| **Amino Acid Metabolism** | N^2^-Succinoylarginine | 274.12777 | 14.83 | C_10_H_18_N_4_O_5_ | -2.23 | | -6.37 | 3.56E-04 |
|  | Hydroxyphenylpyruvic acid | 180.04219 | 7.95 | C_9_H_8_O_4_ | 2.11 | | 1.06 | 2.14E-03 |
| **Lipid Metabolism** | PG 32:2 | 718.47857 | 2.96 | C_38_H_71_O_10_P | -2.17 | | -1.76 | 1.21E-05 |
|  | PG 30:1 | 692.46355 | 2.96 | C_36_H_69_O_10_P | -7.07 | | -7.07 | 9.36E-05 |
|  | PG 34:4 | 742.47703 | 2.94 | C_40_H_71_O_10_P | -6.75 | | -3.36 | 8.99E-06 |
|  | PG 32:0 | 722.50989 | 2.95 | C_38_H_75_O_10_P | -8.35 | | -4.25 | 3.79E-05 |
|  | PS 32:0 | 735.50525 | 2.97 | C_38_H_74_NO_10_P | -7.22 | | -1.47 | 9.85E-05 |
|  | PG 34:1 | 748.52584 | 2.94 | C_40_H_77_O_10_P | -1.98 | | -1.08 | 3.62E-05 |
| **Uniquely Changed Metabolites in the PI^–^ group** | | | | | | | | |
| **Amino Acid Metabolism** | 2-Oxosuccinamate | 131.02181 | 8.89 | C_4_H_5_NO_4_ | 1.43 | | -0.66 | 1.28E-03 |
|  | Arginine | 174.11166 | 25.41 | C_6_H_14_N_4_O_2_ | 1.22 | | -0.01 | 1.89E-03 |
|  | Methionine | 149.05102 | 11.11 | C_5_H_11_NO_2_S | 1.07 | | -0.44 | 3.43E-04 |
|  | Hydroxymethylphosphonate | 111.99257 | 14.16 | CH_5_O_4_P | 1.62 | | -1.27 | 1.32E-03 |
|  | N^2^-Succinyl-L-ornithine | 232.10594 | 13.30 | C_9_H_16_N_2_O_5_ | 1.22 | | -0.27 | 1.28E-03 |
|  | N-Acetyl-L-glutamate 5-semialdehyde | 173.06879 | 7.13 | C_7_H_11_NO_4_ | 1.04 | | -0.70 | 1.55E-03 |
|  | N-Acetylcitrulline | 217.10629 | 12.03 | C_8_H_15_N_3_O_4_ | 1.12 | | -0.15 | 4.15E-03 |
|  | 2-hydroxysuccinamate | 133.03753 | 14.77 | C_4_H_7_NO_4_ | 1.18 | | -0.13 | 2.15E-03 |
|  | beta-Alanyl-L-arginine | 245.14863 | 16.53 | C_9_H_19_N_5_O_3_ | 1.09 | | -0.62 | 2.44E-04 |
|  | Tryptophan | 204.08998 | 11.32 | C_11_H_12_N_2_O_2_ | 1.18 | | -0.35 | 3.53E-03 |
|  | Asparagine | 132.05354 | 15.19 | C_4_H_8_N_2_O_3_ | 1.01 | | -0.20 | 3.02E-03 |
|  | Histidine | 155.06943 | 14.38 | C_6_H_9_N_3_O_2_ | 1.36 | | -0.09 | 1.99E-03 |
|  | N-Acetyl-hydroxy-L-lysine | 204.11108 | 10.06 | C_8_H_16_N_2_O_4_ | 1.56 | | -0.72 | 1.71E-03 |
|  | 2-Keto-glutaramic acid | 145.03746 | 7.34 | C_5_H_7_NO_4_ | 1.16 | | -0.14 | 3.48E-03 |
|  | Lysine | 146.10549 | 23.08 | C_6_H_14_N_2_O_2_ | 1.42 | | -0.35 | 2.82E-03 |
|  | Phenylpyruvic acid | 164.04734 | 4.35 | C_9_H_8_O_3_ | 1.66 | | 0.67 | 6.90E-03 |
|  | Phenylalanine | 165.07901 | 9.64 | C_9_H_11_NO_2_ | 1.10 | | -0.34 | 3.95E-03 |
|  | Tyrosine | 181.07390 | 12.80 | C_9_H_11_NO_3_ | 1.03 | | -0.33 | 2.15E-06 |
|  | Leucine | 131.09461 | 10.21 | C_6_H_13_NO_2_ | 1.02 | | -0.34 | 3.82E-03 |
|  | Serine | 105.04270 | 15.75 | C_3_H_7_NO_3_ | 1.66 | | -0.37 | 3.56E-04 |
| **Carbohydrate Metabolism** | N-acetylneuraminate | 309.10616 | 12.95 | C_11_H_18_NO_9_ | 1.00 | | -0.33 | 1.56E-04 |
|  | Ascorbate | 176.03206 | 15.77 | C_6_H_8_O_6_ | 1.22 | | -0.11 | 3.40E-03 |
|  | Fructose | 180.06338 | 12.83 | C_6_H_12_O_6_ | 1.10 | | -0.09 | 1.79E-03 |
|  | Gluconic acid | 196.05822 | 14.05 | C_6_H_12_O_7_ | 1.05 | | -0.01 | 1.64E-02 |
|  | Altronate | 196.05827 | 12.56 | C_6_H_12_O_7_ | 1.22 | | 0.55 | 8.43E-04 |
|  | Gluconolactone | 178.04774 | 10.80 | C_6_H_10_O_6_ | 1.04 | | 0.28 | 8.35E-05 |
| **Lipid Metabolism** | 2-C-Methyl-D-erythritol 4-phosphate | 216.04005 | 14.24 | C_5_H_13_O_7_P | 1.82 | | -0.75 | 1.76E-03 |
|  | N-Methylethanolaminium phosphate | 155.03495 | 14.17 | C_3_H_10_NO_4_P | 3.14 | | -2.22 | 2.87E-03 |
|  | PE 32:1 | 689.49889 | 3.22 | C_37_H_72_NO_8_P | -2.22 | | -0.83 | 2.65E-05 |
|  | PS 34:0 | 763.53656 | 2.95 | C_40_H_78_NO_10_P | -1.86 | | -0.98 | 2.74E-05 |
|  | PG 34:2 | 746.51024 | 2.94 | C_40_H_75_O_10_P | -1.80 | | -0.88 | 3.41E-05 |
|  | PE 34:1 | 717.53054 | 3.21 | C_39_H_76_NO_8_P | -1.39 | | -0.34 | 1.34E-03 |
| **Nucleotide Metabolism** | Ureidoisobutyric acid | 146.06916 | 12.63 | C_5_H_10_N_2_O_3_ | 1.03 | | -0.08 | 3.00E-03 |
|  | Hypoxanthine | 136.03849 | 9.81 | C_5_H_4_N_4_O | 1.11 | | -0.43 | 1.74E-03 |
|  | Deoxyadenosine | 251.10186 | 10.85 | C_10_H_13_N_5_O_3_ | 1.07 | | -0.42 | 4.49E-04 |
|  | Cytidine | 243.08537 | 11.57 | C_9_H_13_N_3_O_5_ | 1.12 | | -0.30 | 2.51E-03 |
| **Energy, glycan and secondary metabolism** | Trimethylallantoin | 200.09097 | 12.51 | C_7_H_12_N_4_O_3_ | 1.16 | | -0.43 | 1.70E-03 |
|  | Deoxyguanidinoproclavaminic acid | 228.12226 | 14.26 | C_9_H_16_N_4_O_3_ | 1.45 | | -0.22 | 1.04E-02 |
|  | CMP-3-deoxy-D-manno-octulosonate | 543.10924 | 14.76 | C_17_H_26_N_3_O_15_P | 1.18 | | 0.09 | 2.69E-03 |
|  | Dihydrobiopterin | 239.10195 | 9.84 | C9H_13_N_5_O_3_ | 1.20 | | -0.06 | 3.30E-03 |
|  | 2-(Hydroxymethyl)-3-(acetamidomethylene)succinate | 217.05865 | 12.77 | C_8_H_11_NO_6_ | 1.12 | | -0.29 | 1.03E-02 |
| **Uniquely Changed Metabolites in the PI^+^ group** | | | | | | | | |
| **Amino Acid Metabolism** | N-Acetylglutamic acid | 189.06376 | 14.02 | C_7_H_11_NO_5_ | 0.08 | | -1.13 | 1.11E-03 |
| **Carbohydrate Metabolism** | 5-Dehydro-4-deoxy-D-glucarate | 192.02708 | 16.00 | C_6_H_8_O_7_ | 0.18 | | -1.34 | 3.68E-04 |
| **Lipid Metabolism** | sn-glycero-3-PE | 215.05592 | 15.20 | C_5_H_14_NO_6_P | -0.82 | | -1.43 | 3.29E-04 |
|  | PE 34:0 | 743.54603 | 3.20 | C_41_H_78_NO_8_P | -0.12 | | 1.06 | 5.27E-05 |
